# Actinomyces findings in tonsillectomy specimens- comparison between adults and pediatrics populations

**DOI:** 10.1007/s00405-025-09481-y

**Published:** 2025-06-25

**Authors:** Yotam Aharon Shiner, Shoham Caplan, Ilana Doweck

**Affiliations:** 1 Department of Otolaryngology, Head and Neck Surgery, Lady Davis Carmel Medical Center, Haifa, Israel; 2 The Ruth and Bruce Rappaport Faculty of Medicine, Technion Israel Institute of Technology, Haifa, Israel

**Keywords:** Actinomyces, Actinomycosis, Tonsillectomy, Tonsillitis, Obstructive Sleep Apnea

## Abstract

**Background:**

Tonsillectomy is a common surgical procedure primarily performed for obstructive sleep apnea (OSA) and recurrent acute tonsillitis. Actinomyces are gram-positive, anaerobic bacteria commonly found in the oral cavity and known to form part of the human microbiome, occasionally associated with localized infections. While *Actinomyces* bacterial colonization in tonsils has been previously studied, its correlation with surgical indications and outcomes remains unclear.

**Objectives:**

The study aimed to investigate the relationship between *Actinomyces* colonization in tonsillar specimens and surgical indications, and to examine whether *Actinomyces* presence influences tonsillectomy outcomes, specifically post-operative hemorrhage and hospital stay.

**Methods:**

This retrospective cohort study analyzed electronic medical records from Lady Davis Carmel Medical Centre between 2011 and 2021. The study included 1,333 patients who underwent extra-capsular tonsillectomies. Patient demographics, surgical indications, length of hospital stay, post-surgical complications, and pathological reports were collected and analyzed using IBM SPSS Statistics 28.0.

**Results:**

The study population consisted of 760 (57%) males and 573 (43%) females, with an age range of 0.98 to 67.5 years (mean 9.1 years). *Actinomyces* was present in 13.2% of specimens from patients with infectious indications compared to 4.5% in patients with obstructive indications (*p* < 0.001). A statistically significant age difference was observed between patients with (17.78 years) and without (8.55 years) *Actinomyces*. Multivariate analysis revealed that age was the most significant predictor of *Actinomyces* presence (OR 1.047, *p* < 0.0001).

**Conclusions:**

The study found a significant correlation between Actinomyces presence, older age, and infectious surgical indications. While Actinomyces colonization did not demonstrate a direct influence on surgical complications, the findings suggest potential clinical relevance that merits further research to elucidate the bacterium's role in tonsillar pathology.

## Introduction

Tonsillectomy remains the second most prevalent pediatric surgical procedure in the United States, with primary indications being obstructive sleep apnea (OSA) and recurrent acute tonsillitis in the pediatric population. These conditions significantly impact patients and healthcare systems, resulting in frequent hospital visits, extended hospital stays, school absences, and lost workdays for caregivers. Sleep-disordered breathing, particularly in children, often leads to diminished academic and intellectual achievements [1]. Tonsillectomy, typically performed in conjunction with adenoidectomy, has demonstrated efficacy in improving behavioral and cognitive outcomes while reducing healthcare system costs. The procedure has shown particular effectiveness in decreasing infection recurrence rates during the two years following surgery, reducing antibiotic consumption, lowering the frequency of primary care visits and hospital referrals, and substantially improving or completely resolving OSA symptoms in affected patients.

Less common indications for tonsillectomy include recurrent peritonsillar abscesses, aneurysms (secondary to sleep-disordered breathing), suspected malignancy, dental arch malocclusion, speech disturbances, primary snoring and halitosis [2].

Tonsillectomy, like all surgical interventions, carries potential risks. The primary concern is intraoperative or postoperative hemorrhage [1, 3, 4]. Significant hemorrhage may necessitate additional hospitalizations and surgical intervention under general anesthesia to achieve hemostasis. Other complications include general anesthesia-related issues, dental trauma, oral mucosa or pharyngeal injury, laryngeal edema and spasm and in rare cases, death. Adults experience more complications than children. Within the pediatric population, certain groups show increased risk, including patients with craniofacial anomalies, trisomy 21, cerebral palsy, and neuromuscular disorders.

While the literature extensively covers pediatric tonsillectomy, studies on adult populations are comparatively limited. In adults, the primary indications differ, focusing more on recurrent infections, Peritonsillar abscesses, suspected malignancies, and halitosis.

Routine histopathological examination of specimens is often performed to rule out malignancy or granulomatous disease [5].

*Actinomyces* are slow-growing, non-spore-forming, and non-acid-resistant anaerobic bacteria with a branched filamentous shape. Of the six human-pathogenic types, *Actinomyces israelii* and *Actinomyces naeslundii* are the most common [6].

The prevalence rate of *Actinomyces* in tonsillar specimens ranges in different reports from 1.3% to 48% [7, 8]. In the oral cavity, *Actinomyces* has been found in carious dentin, dental plaque, on the gingival surface, and in tonsillar crypts [9–13]. *Actinomyces* species are naturally found in the human oropharynx, gastrointestinal tract, and urogenital tract as commensal organisms. Infections caused by these bacteria are primarily endogenous, and occur when the integrity of the tissue is compromised due to a mucosal or skin lesion, allowing the microorganisms to invade local structures and become pathogenic. The infection can lead to the formation of granulomatous tissue, reactive fibrosis, necrosis, abscesses, draining sinuses, and fistulas [14, 15].

### Study objectives

Our primary aim is to investigate the correlation between *Actinomyces* colonization in tonsillar specimens and surgical indications (primarily obstructive versus infectious), as previous studies have yielded inconclusive results. Understanding this relationship could improve patient selection and potentially lead to less invasive treatment approaches when *Actinomyces* is detected preoperatively. Additionally, we aim to examine whether *Actinomyces* presence influences tonsillectomy outcomes, particularly regarding post-operative hemorrhage and length of hospital stay.

### Study design and setting

This retrospective cohort study is based on electronic medical records (EMR) of Lady Davis Carmel Medical Centre, Haifa, Israel. Diagnoses were determined using International Clinical Diagnosis version 9 (ICD-9) specific codes. All the procedures were performed by a small group of surgeons, all of them trained in the same method and used cold steel dissection technique. Monopolar diathermy was used only if needed for hemostasis. All tonsillectomy specimens were routinely sent for histopathological assessment, regardless of any clinical suspicion or specific indication.

The presence and identification of *Actinomyces* colonies were performed by senior pathologists, using conventional staining techniques of histopathological specimens.

### Data collection

The following data were collected from the Lady Davis Carmel Medical Center  medical records:

- Patient demographics (e.g., age and sex), Indication for surgery, length of hospital stay, events of post-surgical complications, Pathological reports of tonsillar specimens.

### Study population

All patients who underwent extra-capsular tonsillectomies with subsequent pathological review of tonsils between 2011 and 2021 were identified using the EMR. Medical records were reviewed to verify diagnoses and retrieve data.

### Ethical compliance

The study was performed following approval and in accordance with the ethical standards of the institutional review board of Lady Davis Carmel Medical Center and the 1964 declaration of Helsinki and its later amendments. The manuscript has been prepared with reference to the STROBE checklist for cohort studies.

### Statistical analysis

Continuous variables are presented as means with standard deviations (SD) or medians and inter-quartile range (IQR). Categorical variables are presented as numbers and proportions. Differences in demographic and clinical characteristics between recurrent and non-recurrent cases were analyzed using the Levin and Chi-square test. Multivariate Cox proportional hazard analysis was used to identify independent prognostic factors of recurrent AM. The Hazard Ratio (HR) is presented with a 95% Confidence Interval. All statistical analyses were performed using IBM SPSS Statistics 28.0. For all analyses, *p* < 0.05 (for two-tailed tests) was considered statistically significant.

## Results

Our database identified 1333 patients, comprising 760 (57%) males and 573 (43%) females. The age range was 0.98 to 67.5 years, with an average age of 9.1 years. Clear surgical indications were retrieved for 1077 patients, and specific information regarding the presence or absence of *Actinomyces* was available for 1061 patients. 306 (28%) patients were operated on due to infectious indications; 771 (72%) patients were operated on due to obstructive indications (Table 1).

**Table 1:** Descriptive statistics of the study population

Parameter	*N* (%)	Total
Sex		*N* = 1333
Male	760 (57%)	
Female	573 (43%)	
Age (years)		*N* = 1333
mean ± SD	9.1 ± 10.32	
Range	0.98–67.5	
Indication for surgery		*N* = 1077
Obstructive	771 (72%)	
Infectious	306 (28%)	
Presence of Actinomyces in specimen		*N* = 1061
Positive	74 (6.97%)	
Negative	987 (93.03%)	
Post-surgical Complications		*N* = 1077
Positive	30 (2.8%)	
Negative	1047 (97.2%)	

Complications were described in 30 (2.7%) patients. A statistically significant difference (*p* < 0.001) was observed between the average age of patients with *Actinomyces* (17.78 years) and those without it (8.55 years). *Actinomyces* was present in 13.2% of specimens from patients operated on due to infectious diagnoses (40/303 patients), compared to 4.5% of specimens from patients operated on for obstructive diagnoses (34/758 patients) This difference is statistically significant (*p* < 0.001). No statistically significant difference in *Actinomyces* presence was found between the sexes. The average length of hospital stay was similar (2.18 days) for patients with and without *Actinomyces* presence. In a multivariate analysis including age, sex, and surgical indication, the most significant predictor of *Actinomyces* presence was patient age (OR 1.047, *p* < 0.0001). The rate of *Actinomyces* presence among patients without complications was 6.8%, compared to 13.3% among those who experienced complications. However, this difference is not statistically significant (*p* = 0.165). No significant correlation was found between sex and complication rate (*p* = 0.153). The average age of patients without complications (8.92 ± 10.1 years) was significantly lower than those with complications (19.8 ± 14.6 years; *p* < 0.001). The complication rate for patients operated on for infectious indications (6.2%) was significantly higher than for obstructive indications (1.4%) (*p* < 0.001) (Tables 2, 3).

**Table 2:** Univariate analysis – presence of actinomyces

	Actinomyces positive	Actinomyces negative	*p*-value
Mean Age (years)	17.78 ± 14.58	8.55 ± 9.7	< 0.001
Mean length of stay (days)	2.18 ± 0.795	2.18 ± 0.747	0.91
Complication			
Negative (*n* = 1031)	70 (6.8%)	961 (93.2%)	0.165
Positive (*n* = 30)	4 (13.3%)	26 (86.7%)	
Indication			
Obstructive (*n* = 758)	34 (4.5%)	724 (95.5%)	< 0.001
Infectious (*n* = 303)	40 (13.2%)	263 (86.8%)	
Gender			
Male (*n* = 606)	43 (7.1%)	564 (93.9%)	0.858
Female (*n* = 455)	31(6.8%)	424 (93.2%)

**Table 3:** Univariate analysis – complications

	Complications Occurred	Complication free	*p*-value
Mean Age (years)	19.8 ± 14.6	8.92 ± 10.1	< 0.001
Indication			
Obstructive (*n* = 771)	11 (1.4%)	760 (98.6%)	< 0.001
Infectious (*n* = 306)	19 (6.2%)	263 (93.8%)	
Gender			
Male (*n* = 617)	30 (3.4%)	587 (96.6%)	0.153
Female (*n* = 460)	9 (2%)	424 (98%)	

In multivariate analysis, the most significant independent risk factors for complications were:

1. Age at the time of surgery (OR 1.044, *p* = 0.004)
2. Sex (male predominance, OR 2.354, *p* = 0.04)
3. Indication for surgery (infectious predominance, OR 2.52, *p* = 0.054, borderline significance)

The analysis revealed an additional risk of 4.4% for complications with each year of age. Male patients had a 2.35 times higher complication rate. Infectious indications showed a 2.52 times increased likelihood of complications, though this was on the margin of statistical significance (Table 4).

**Table 4:** Multivariate Analysis of Risk Factors for Complications

	Odds Ratio	Range	*P*-Value
Age	1.044	1.014–1.075	**0.004**
Indication (infectious/obstructive)	2.521	0.983–6.475	0.54
Gender (Male)	2.354	1.041–5.322	**0.04**
Presence of Actinomyces	0.951	0.301–3.006	0.932

## Discussion

In this study, we found a significant association between the presence of Actinomyces, older age, and an infectious indication for tonsillectomy. However, while Actinomyces presence alone was not an independent risk factor for surgical complications, older age and an infectious indication were identified as significant risk factors. This suggests that, in certain cases, Actinomyces presence may indicate a higher likelihood of surgical complications when combined with these factors. It is important to note that, since all procedures and post-operative care were performed within a single institution by the same team of surgeons, the potential for confounding by variations in surgical technique, surgeon experience, and care protocols is reduced.

As opposed to our findings, Bhargava et al. reported a higher rate of *Actinomyces* presence in hypertrophic tonsils rather than those operated for recurrent infections [7]. Daneshmandan et al. have also pointed out an association with tonsillar hypertrophy [16]. Various theories were hypothesised in order to explain the connection to the tonsillar size, one of which was that the *Actinomyces* cause lymphoid hyperplasia of the tonsillar tissue, however, the theory has been proven. Another explanation for the higher rate of *Actinomyces* presence in the tonsils that were operated on due to obstructive indications might be the fact that patients who suffer from recurrent tonsillitis, are often treated with numerous courses of antibiotic therapy, sometimes with broad-spectrum antibiotic agents, which might reduce or eliminate the *Actinomyces* colonization in the tonsils.

Other authors identified a positive correlation between patient age and *Actinomyces* presence but found no connection to tonsillar hypertrophy [6, 17]. Several different research groups observed higher *Actinomyces* prevalence in older patients rather than younger children, and some of them even managed to establish a better correlation in patients with sleep-disordered breathing compared to those with recurrent infections [8, 9, 18]. In concordance with our findings, In a report by Kansu, a higher prevalence of *Actinomyces* was reported in male specimens. A correlation between enlarged tonsils in both pediatric and adult populations was also noted [13].

Different attempts to postulate a correlation between *Actinomyces* and recurrent tonsil infections have yet to yield a definitive conclusion. In a histological and bacteriological study of 129 pairs of tonsils from patients with recurrent acute tonsillitis, Gaffney et al. concluded that *Actinomyces* does not have a causal role in recurrent acute tonsillitis.

In a large retrospective study of 1820 tonsillectomies by Aydin et al., there was also no correlation between the clinical diagnosis of tonsillar disease and the presence of actinomycosis both in adult and pediatric patients [19].

Given the complexity of our findings, further research is needed to better understand the role of Actinomyces in surgical outcomes. Future studies should focus on larger, more diverse cohorts to validate these associations and explore the potential causal relationship between Actinomyces presence and post-operative complications.

## Data Availability

The data of this study are available from the records of the Lady Davis Carmel Medical Center, but restrictions apply to the availability of these data, which were used under the license for the current study and are not publicly available.

## Abbreviations

OR: Odds Ratio
IQR: Inter-Quartile Range
SD: Standard Deviation
